# Comparison between ammonium formate and ammonium fluoride in the analysis of stratum corneum lipids by reversed phase chromatography coupled with high resolution mass spectrometry

**DOI:** 10.1038/s41598-023-50051-1

**Published:** 2024-01-02

**Authors:** Miriam Maiellaro, Grazia Bottillo, Alessia Cavallo, Emanuela Camera

**Affiliations:** Laboratory of Cutaneous Physiopathology, San Gallicano Dermatological Institute - IRCCS, Via Elio Chianesi 53, 00144 Rome, Italy

**Keywords:** Lipidomics, Mass spectrometry

## Abstract

Lipids are key constituents of the barrier function in the human stratum corneum (SC), which is the outermost layer of the epidermis and amenable to non-invasive sampling by tape stripping. The three major lipid classes in the SC, i.e., ceramides, fatty acids, and cholesterol, present equimolar concentration. Liquid chromatography coupled with mass spectrometry (LCMS) is elective in profiling lipids in the SC in both positive and negative ion modes. Nevertheless, the latter one allows for the simultaneous detection of the three major epidermal components of the SC. Determination of ceramides in the SC poses analytical challenges due to their wide range of structures and concentrations especially in the case of limited sample amounts. Ammonium formate is a commonly used modifier added to the mobile phase to assist ionization. However, it introduces uncertainty in the identification of ceramides when operating in negative ion mode, even with high resolution MS. We tested the advantages of using fluoride in the lipid profiling of SC and unambiguous identification of ceramides subclasses. The use of fluoride enhanced the ionization of ceramides, regardless the specific substructure, solved misidentification issues, and was successfully applied to the simultaneous detection of all three lipid classes in the human SC.

## Introduction

Lipidomics is the comprehensive study of lipids, which are hydrophobic molecules belonging to a wide range of molecular classes. It involves the identification, quantification, and characterization of the full complement of lipids present in biological systems such as cells, tissues, and organs. Lipidomics aims to provide a detailed understanding of the structure, function, and regulation of lipids, and how they are involved in various physiological and pathological processes, including metabolism, inflammation, and diseases. Lipids are essential for several functions deployed by human skin^1,2^. The permeability barrier residing in the stratum corneum (SC), which is the outermost layer of the epidermis, is a key skin function granted by a specific lipid milieu that regulates water loss and protects against environmental stressors. The fundamental components of the permeability barrier are ceramides (CERs), free fatty acids (FFAs), cholesterol and its metabolites. The mole proportion of the three lipid classes is balanced, as each one accounts for one third of the total. In contrast, in the skin, the complexity of molecular structures is extremely high for CERs, intermediate for FFAs, and relatively low for cholesterol and its congeners. The SC contains approximately 50% CERs, 15% free fatty acids, and 25% cholesterol and cholesterol sulfate (CHS) as weight/weight%^3,4^. CERs account for a key subclass in the sphingolipid metabolism. Structurally, CERs are composed of sphingoid long chain base (LCB) covalently bound via amide linkage to a fatty acid (FA). These two moieties can vary in length, hydroxylation, number, position and geometry of unsaturation and possible presence of a functional group^5,6^. Structures and nomenclature for CERs in mammals are extensively explained in Kawana et al.^6^. Combining the various LCBs with different FFAs ensues an extremely complex and diverse array of species, which include isobars and isomers. CERs are ubiquitous in human body but, in comparison with other human tissues, in the SC, CERs display an exceptional variety of structures and wide dynamicity of their concentration^7^. The most represented LCB moieties among CERs in the human SC are dihydrosphingosine (DS), sphingosine (S), phytosphingosine (P), and 6-hydroxysphingosine (H). Recently, CERs containing minor LCBs derived from non-canonical biosynthetic pathways, such as 1-deoxysphinganine, 1-deoxy-sphingosine and 4,14-sphingadienine (SD), have been described. LCBs mainly have a chain length of C_18_–C_20_, but chain length can vary from 12 to 26 carbon atoms. FAs chain length can vary from C_14_ to C_26_, but longer chains are not uncommon. FAs can be non-hydroxy (N), α-hydroxy (A), ω-hydroxy (O), esterified ω-hydroxy (EO). When a CER contains a FA hydroxylated at the ω position of the carbon chain, it can be additionally esterified to another FA forming O-acylceramides or it can be combined with proteins forming protein-bound CERs^8,9^. β-hydroxy (B) FAs occur rarely^5,7^. Each ceramide class is designated by a combination of the abbreviations of the FA and LCB, respectively, e.g., N FAs combined with S LCB form the Cer[NS] class.

Comprehensive analysis of CERs is extremely important due to their regulatory role, biochemical functions, variety of chemical structures, and the wide range of concentrations.

Liquid chromatography (LC) is the mainstay of the CERs separation. Normal phase LC (NPLC) operates CERs separation according to their hydrophilic functionalities, whereas separation by reversed phase LC (RPLC) is linked to the CERs hydrophobic properties^5^.

RPLC is preferred because of its elevated separation efficiency. Electrospray ionization (ESI) is extensively used in the analysis of CERs in mass spectrometry (MS) coupled to LC (LCMS). Because of their chemical structure, CERs are detectable in both positive (+ ESI) and negative (− ESI) ion modes. In + ESI mode, CERs are detected in their protonated form, i.e., as [M + H]^+^ ion, which can undergo neutral loss (NL) of the water molecule yielding the [M + H-H_2_O]^+^ ion^10^. In the presence of alkaline ions (Alk^+^), i.e., Li, K, and Na, the [M + Alk]^+^ ions are observed. In -ESI mode, CERs form the [M − H]^−^ deprotonated ion. Due to the presence of anions deriving from salts, i.e., formate, acetate, halogens, which are altogether indicated as X, the [M + X]^−^ adducts occur with an abundance dependent upon the chemical features of the different CERs subclasses. The most commonly used chloride^11^, formate and acetate anions promote the formation of [M + Cl]^−^, [M + HCOO]^−^, [M + CH_3_COO]^−^ adduct ions, respectively^12–14^. CERs ionized in either polarity generate characteristic spectra when fragmented under appropriate conditions. The rules of fragmentation have been largely characterized in both polarities providing enough spectral information to derive structural characterization. Generally, the + ESI mode is used for the identification and quantification of CERs, while the − ESI mode for the acquisition of the expression profiles of the different subspecies^5,15^. The choice of the polarity is also based on the availability of MS analysers with low or high mass resolution. The low resolution triple quadrupole MS is preferentially used in the CERs profiling by multiple reaction monitoring (MRM) along with retention time (RT), according to well established libraries of precursor and associated fragment ions defined in + ESI^1,16^. High resolution mass analysers, which range from single stage, i.e., TOF and Orbitrap, to hybrid high resolution MS (HRMS), e.g., Q-TOF, have the advantage of excellent accuracy (2 ppm or lower). Profiles of CERs analysed according to the accurate mass of the molecular or pseudo-molecular ion are acquired either in + ESI or − ESI. Hybrid HRMS present multiple operational modes, such as full scan, product or precursor ion scan, NL scan, selected reaction monitoring (SRM) and MRM, which are very helpful to validate methods and to identify known and unknown molecules. Ideally, a method applied to the analysis of epidermal lipids allows the detection of cholesterol-related compounds, and FFAs simultaneously to CERs. The common feature shared by members of the three major classes of epidermal lipids, is the ionization by deprotonation or by adduction to organic and inorganic anions.

Thus, we planned to explore − ESI mode to acquire lipidomics data in the interest of the simultaneous detection of the main epidermal lipid classes in the SC. Formic acid (HCOOH) and ammonium formate (NH_4_COOH) are the most commonly used additives in − ESI RPLC analysis^17^. However, the use of formate generates some ambiguities that hampers identification, especially for CERs that pose additional challenge due to their elemental composition and multitude of structures. To limit misidentification that may alter the biological interpretation of results, we explored the advantages of replacing formate with fluoride by using the ammonium fluoride (NH_4_F) salt, which has become a suitable eluent additive in metabolomics studies by LCMS methods^17–19^. There is evidence of NH_4_F signal-enhancing effect^20,21^, especially for the determination of steroids^22^. Additionally, NH_4_F assisted ionization improves sensitivity through the promotion of the [M − H]^−^ ion and the formation of the [M + F]^−^, and the [M + HF + F]^−^ clusters^18,22^. We propose that NH_4_F is beneficial in the accurate identification and quantitation of CERs in human SC and allows the simultaneous detection of FFAs and cholesterol sulfate.

## Materials and methods

### Chemicals

ULC-MS grade acetonitrile, ethanol, 2-propanol, methanol and ultra-pure water were purchased from Biosolve (Chimie SARL, Dieuze, France; BV, Valkenswaard, Netherlands). Chloroform was purchased from Carlo Erba (Milan, Italy). NH_4_COOH, NH_4_F, and butylated hydroxytoluene (BHT) were purchased from Sigma Aldrich (Milan, Italy). The Q-TOF calibration solution was prepared in acetonitrile from Agilent Technologies Tuning mix (HP0321 solution, Agilent Technologies, Santa Clara, USA).

N-palmitoyl-d31-D-erythro-sphingosine (d31-Cer[NS]34:1, MW 569.1), N-[26-oleoyloxy(d9) hexacosanoyl]-d-erythro-sphingosine (Cer[EOS] (d18:1/26:0/18:1(d9), MW 967.6) and *N*-[26-oleoyloxy hexacosanoyl]-d-erythro-sphingosine (Cer[EOS] (d18:1/26:0/18:1), MW 958.6) were purchased from Avanti Polar Lipids (Alabaster, USA). Deuterated cholesterol sulfate sodium salt (d7-CHS, MW 495.3) and Hexadecanoic-9,9,10,10,11,11,12,12,13,13,14,14,15,15,16,16,16-d17 acid (d17-PA, MW 273.5) were purchased from CDN Isotopes Inc., (Pointe-Claire, Canada). Stock solution of the internal standard (iSTD) mixture of deuterated compounds was prepared in 2-propanol with the following concentrations: d7-CHS 40 µM, d17-PA acid 80 µM, d31-Cer16:0 10 µM. d9-CER[EOS] and CER[EOS] mixture (2 µM each in chloroform/methanol (2:1)) was introduced to confirm identification of O-acylceramides.

### Sampling of stratum corneum, lipid extraction and preparation of pooled samples

SC samples were collected in a previous study conducted in 44 healthy volunteers after informed consent was given. The study followed the principles of the Declaration of Helsinki and was approved by the Institutional Review Board (IRB) of the San Gallicano Dermatological Institute without a formal IRB because no ethical concerns were raised over the use of the SC samples as anonymized material in this study. The donors of SC samples comprised 17 male and 27 female adult participants with a mean age 35.5 ± 14.1, and 35.7 ± 16.3, respectively. Adhesive tapes (Corneofix® CF 20, Courage + Khazaka electronic GmbH, Köln, Germany) were used to collect SC samples from facial areas, which represented the sebaceous gland (SG) rich (SGR) area and from arm, which was referred to as the SG poor (SGP) area. Two tapes have been used to sample the SC from the same site, following removal of the first three SC layers with a patch then discarded. Once the sampling step was completed, the patches were stored at a temperature of − 80 °C until extraction. To obtain lipid extracts, each adhesive patches was positioned inside a 60 × 15 mm PE-LD Petri’s dish (PD) (CELLSTAR, Greiner Bio-One GmbH, Frichenhausen, Germany) with the adhesive side upwards. 1 mL of 2-propanol containing the deuterated iSTD mix and methanol containing BHT 1.2 mM was added to the samples to control the analytical process and to quantitate the relative abundance of detected lipids. Based on preliminarily optimized conditions of the sample processing, the adhesive patches were incubated at 37 °C for 10 min in thermostatic oven to favor lipid extraction. Then, the content of each dish was transferred into an amber glass vial. 50 µL of 2-propanol were added to the PD to collect the residues of the extraction mixture; this volume was then pooled with the previous solution. The final extract was filtered through the Agilent’s system Captiva Vacuum (Agilent Technologies, Santa Clara, USA) with a pore size of 0.2 µm connected to a vacuum pump. The filtrate was collected in graduated glass vials and brought to the volume of 1.5 mL with 2-propanol.

Quality control (QC) samples were generated by pooling equal volumes of extracts of individual samples. Specifically, the SGR QC sample was obtained by unifying 10 µL volumes taken from samples from facial areas. The QC sample representing the SGP areas, was obtained by unifying 10 µL volumes of each extract from SC taken from arm. Both SGR and SGP QCs samples were used to compare effects of analytical conditions and adduct formation, on sensitivity, and reproducibility.

### Liquid chromatography

High Performance LC (HPLC) was performed on an Infinity II 1260 series HPLC system with a degasser, a quaternary pump, a thermostated autosampler set at 6 °C (Agilent Technologies, Santa Clara, USA). For the RP-HPLC separation, the SB-C8 Zorbax RR HT column (Agilent Technologies, Santa Clara, USA), 2.1 × 100 mm, 1.8 µm particle size, with maximal operational backpressure at 600 bar, was used. The column was thermostated at 60 °C in the column compartment.

Elution was performed with a binary gradient of: (A) ultra-pure water containing either 5 mM NH_4_COOH or 0.2 mM NH_4_F and (B) methanol/2-propanol 80/20. When the eluent (A) contained 0.2 mM NH_4_F, this salt was present in the (B) eluent at the same concentration. The gradient for lipid species separation was set as follows: 40% B, 0–2.0 min; 40–99% B, 2.0–36.0 min, 99% B 36.0–46.0 min, 99–40% B 46.0–48.0 min. The flow rate was 0.3 mL/min; the injection volume was 1 µL. The total run time was 48 min. An isocratic hold of 10 min of the initial starting conditions was set for column re-equilibration between each injection. Carryover effect was minimized by needle wash between each injection.

### High resolution mass spectrometry

MS analysis was performed on a 6545 Q-TOF mass spectrometer equipped with an ESI Dual Agilent Jet Stream (AJS) interface (Agilent Technologies, Santa Clara, USA), operating in the − ESI mode. Gaseous nitrogen was used for both nebulization and desolvation processes. Gas temperature was set at 200 °C with a flow rate of 12 L/min. The gas pressure in the nebulizer was set at 40 psi, the temperature, and the flow rate of the sheath gas were set at 350 °C, and 12 L/min, respectively. The capillary voltage parameter was 4000. The fragmentor, and the skimmer voltage parameter was 120 and 40, respectively. Data independent acquisition (DIA) was adopted in the initial explorative method by means of the all-ions tandem mass spectrometry (MS/MS) mode at multiple collision energies (CEs): 0, 20, and 40 eV. The data dependent acquisition (DDA) was performed by means of targeted MS/MS to confirm the identity of candidate lipids through the interpretation of spectral patterns. The m/z range was 50–1700 amu for both full MS and MS/MS used to acquire the accurate mass spectra at a mass resolving power of 40,000. To enhance accurate mass measurement, a reference solution of two compounds for each additive (m/z 112.9856 and m/z 966.0007 using NH_4_COOH, m/z 119.0363 and m/z 940.0015 using NH_4_F) was vaporized by a separate nebulizer in continuum in the spray chamber.

### LC-HRMS data processing

LC-HRMS data were acquired and deconvoluted using the MassHunter Data Acquisition Software (B.09.00, Agilent Technologies). Chromatographic and spectral investigation was conducted on MassHunter Workstation Qualitative Analysis software (version 10.0, Agilent Technologies); quantitative analysis of candidate epidermal lipid species on QCs was conducted on MassHunter Workstation Profinder (version 10.0, Agilent Technologies). LipidCreator Lifs tool (version 1.1.0) was used for in silico spectral library support and CERs comprehensive characterization^23^. The multivariate statistical analysis was performed by the software Agilent MassHunter Mass Profiler Professional (MPP, version 15.1, Agilent Technologies).

## Results

### Effects of ammonium formate on the separation and detection of ceramides

**Figure 1:**
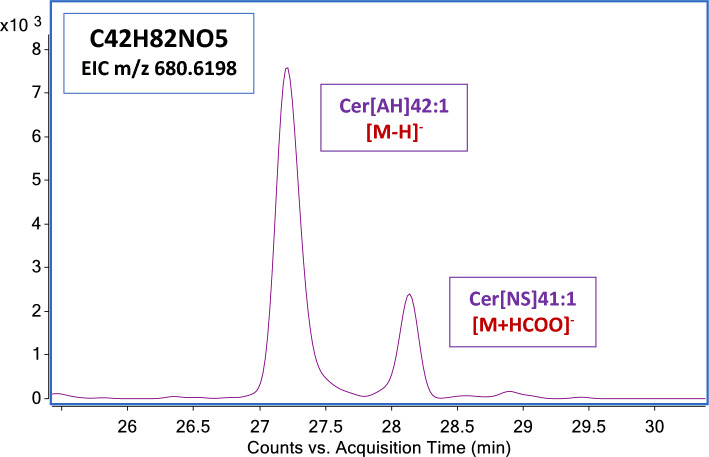
EIC of m/z 680.6198 (C42H82NO5) attesting the presence of two peaks. The first peak (RT 27.320 min) is the [M − H]^−^ ion of the Cer[AH]42:1; the second peak (RT 28.15 min) is the [M + HCOO]^−^ molecular adduct of the Cer[NS]41:1.

Using a gradient elution with ammonium formate as mobile phase modifier at the 5 mM concentration, the formation of the stable anionic adduct [M + HCOO]^−^ was promoted, while the production of the deprotonated molecular ion [M − H]^−^ was less favored. In some cases, the prominent peak due to the formate adduct may interfere with the chromatographic identification of the species of interest. In fact, when operating an extracted-ion chromatogram (EIC) of the m/z 680.6198, corresponding to the [M − H]^−^ ion of Cer[AH]42:1, which has the elemental formula C42H82NO5, using a mass window of 10 ppm, two peaks were observed. Because of the RT and the relative abundance, we supposed that the second peak could be the dominant [M + HCOO]^−^ peak of Cer[NS]41:1, which has the elemental formula C42H82NO5 (Fig. 1).

The empirical formula of the [M + HCOO]^−^ adduct of Cer[NS]41:1 is coincident with the empirical formula of the deprotonated Cer[AH]42:1. Our hypothesis was confirmed by the observation of fragments in the MS/MS spectra (Supplementary Fig. S1). This observation was confirmed to apply to all the CERs members belonging to the respective NS and AH subclasses (Supplementary Table S1). Cer[AP] and Cer[NDS] present the same ambiguity issue: The empirical formula of the [M + HCOO]^−^ adduct generated by Cer[NDS] is indistinguishable from the empirical formula of the [M − H]^−^ ion of the Cer[AP] family members one carbon shorter (Supplementary Table S2). Notwithstanding the coincident accurate mass, RT and MS/MS spectra assisted discrimination of the two anionic forms.

### Comparison between formate and fluoride salts of ammonium on the separation and detection of ceramides by LCMS

**Figure 2:**
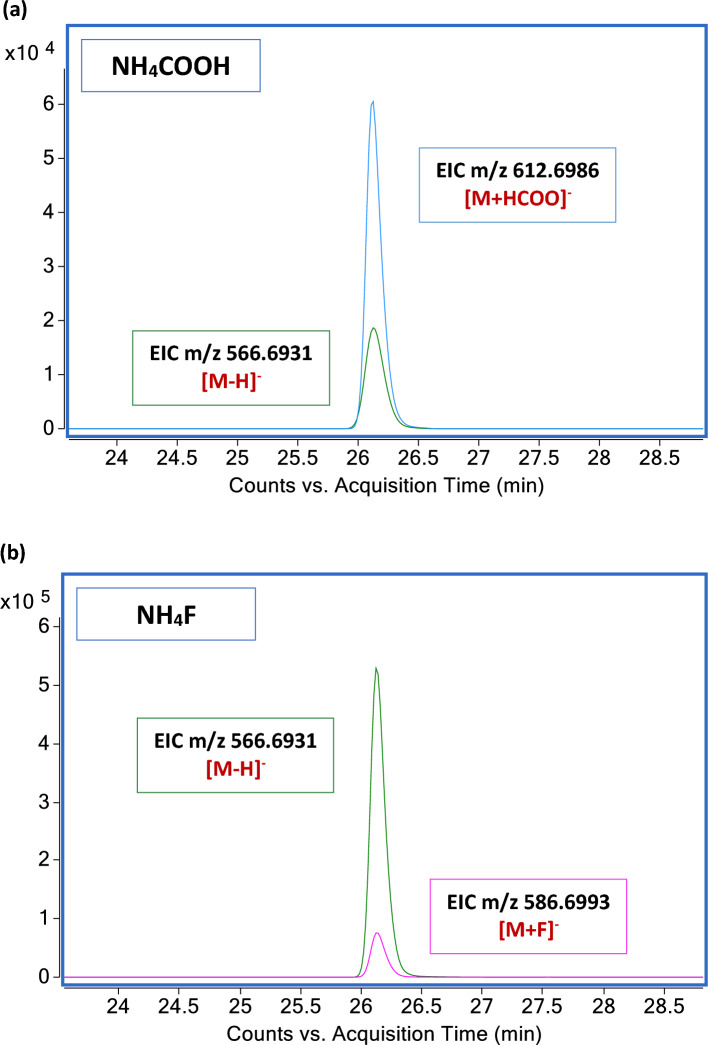
Effects of mobile phase modifiers on the peak intensities of the deuterated ceramide d31-Cer[NS]34:1. (**a**) Negative ionization assisted by NH_4_COOH. Comparison of the intensity of the EIC (m/z 566.6931) and EIC (m/z 612.6986) corresponding to the [M − H]^−^ ion and [M + HCOO]^−^ adduct ion of the d31-Cer[NS]34:1, respectively. (**b**) Negative ionization assisted by NH_4_F. Comparison of the intensity of the EIC (m/z 566.6931) and EIC (m/z 586.6993) corresponding to the [M − H]^−^ ion and [M + F]^−^ adduct ion of the d31-Cer[NS]34:1, respectively. The EICs of the [M − H]^−^, the [M + HCOO]^−^, and the [M + F]^−^ ions are colored in green, blue, and pink, respectively.

To circumvent the confusion deriving from the addition of atoms common to the elemental composition of the CERs lipid class and minimize misidentification errors, we evaluated the effects of adding a different eluent additive. Given to the advantages in the ionization of hydrophobic compounds with NH_4_F ammonium fluoride described in the literature^24^, we explored the effects of adding NH_4_F in our analytical conditions and compared the performance with that obtained with NH_4_COOH. To compare the two chromatographic conditions, which are detailed in the materials and methods, we studied the ionization behavior of the deuterated d31-Cer[NS]34:1 lipid reference. Both modifiers spiked into the mobile phase produced the deprotonated ion [M − H]^−^ and the adduct formed with either formate or fluoride anion provided with the additive. As shown above, in addition to the [M − H]^−^ ion, NH_4_COOH promoted the formation of a stable and intense [M + HCOO]^−^ molecular ion. In contrast, NH_4_F promoted the formation of the [M − H]^−^ ion as a dominant peak opposite to a less intense fluoride-anionic adduct [M + F]^−^ (Fig. 2). Nevertheless, the height of [M − H]^−^ and [M + F]^−^ ions varied among CERs subclasses characterized by different chain length, desaturation degree, and number of hydroxyl-groups.

Comparing the EIC of the [M − H]^−^ ion in the two gradients conditions, RT and the peak shape were similar, but the peak intensity and the peak area were considerably enhanced with NH_4_F. Moreover, fluoride-assisted ionization in negative ion mode caused a notable improvement of the signal to noise ratio (SNR). Comparing the values returned by the MassHunter Qualitative Analysis software for the [M − H]^−^ ion, fluoride (SNR = 8.06e+08) provided a six-fold improvement of the SNR over formate (SNR = 1.24e+08).

To deepen fluoride-mediated negative ionization efficiency, MS/MS spectra obtained with the two different eluent additives were compared using deuterated d31-Cer[NS]34:1 as a lipid reference.

**Figure 3:**
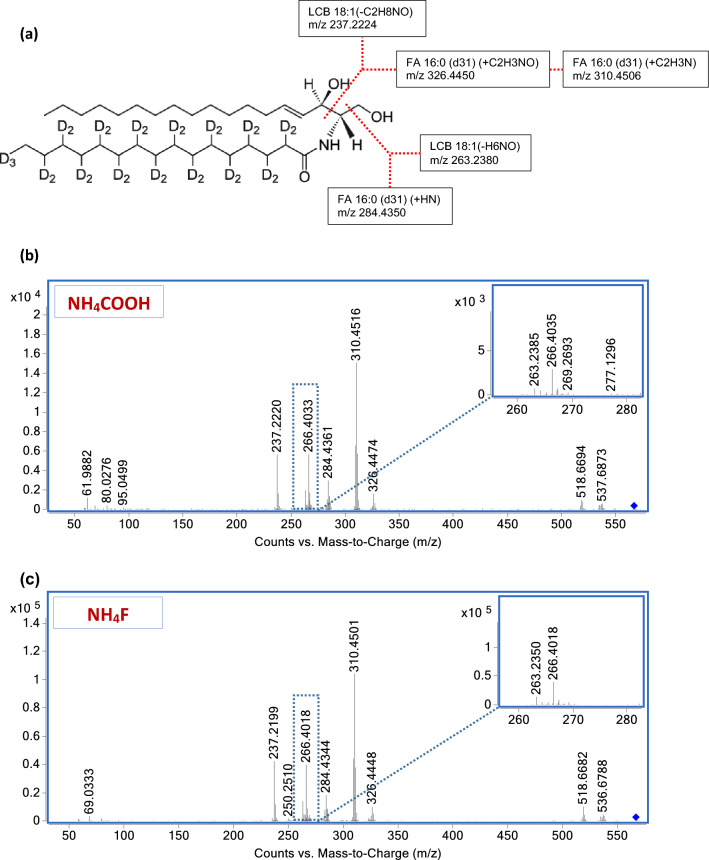
MS/MS spectrum obtained by targeted fragmentation at a CE of 40 eV of deprotonated ion of the deuterated ceramide d31-Cer[NS]34:1. (**a**) Chemical structure and diagnostic fragmentations of LCB and FA. (**b**) NH_4_COOH assisted ionization [M − H]^−^ MS/MS spectrum. (**c**) NH_4_F assisted ionization [M − H]^−^ MS/MS spectrum.

The spectra of both molecular ions at a CE of 20 eV provided poor structural information: The deprotonated molecule fragment was the most abundant, whereas all other fragments produced, showed low signal intensity (Supplementary Fig. S2). The fragmentation pattern of [M + HCOO]^−^ and [M + F]^−^ adduct at a CE of 40 eV was very similar to the corresponding [M − H]^−^ precursor ion, but the diagnostic fragments intensity was definitely lower^18^ (Supplementary Fig. S3). When investigating the fragmentation pattern of the [M − H]^−^ ion promoted by NH_4_COOH and NH_4_F at a CE of 20 and 40 eV, we observed that the fragmentation patterns were similar, but the intensity of the diagnostic fragment was definitely higher when NH_4_F was used. In Fig. 3, d31-Cer[NS]34:1 fragmentation pattern of [M − H]^−^ is reported.

### Ammonium formate versus ammonium fluoride in the LCMS analysis of ceramides in lipid extracts of SC

The advantages of using NH_4_F as eluent additive in the CERs LC methodology were extended to virtually all CERs present in the SC. We focused on CERs derived from the combination of four LCBs, i.e., S, DS, P, and H, and two types of FAs, i.e., N and A. We did not take into account Cer[ADS], whose identification remains challenging despite the optimization of the method, because of their extremely low abundance in the SC and their possible co-elution with Cer[NP] in the applied LC conditions. Cer[NH] and Cer[AS] are isomers, but each subclass is chromatographically resolved as a single peak. Cer[NH] eluted first^5^. In our conditions, Cer[NP] and Cer[AS] co-eluted, but their diagnostic MS/MS fragmentation pattern allowed a confident identification and characterization.

**Figure 4:**
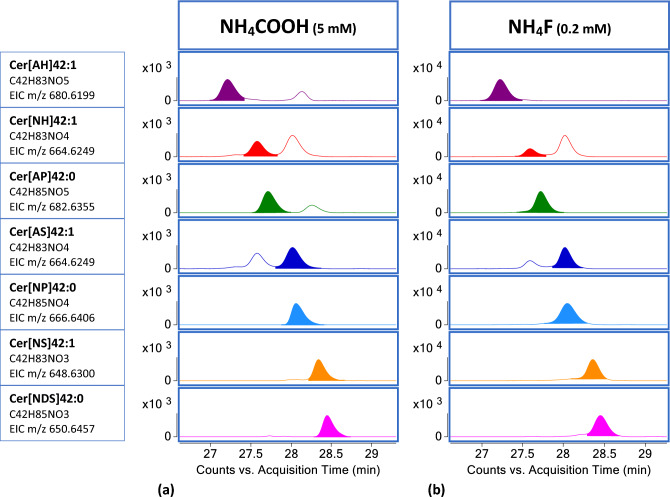
EICs of [M − H]^−^ of CERs with C_42_ total carbon atoms from the seven subclasses detected in the SC. The elution order was as follows: Cer[AH], Cer[NH], Cer[AP], Cer[AS] coeluting with Cer[NP], Cer[NS] and Cer[NDS]. RT and peak shape and intensity are illustrated for the LCMS conditions using (**a**) NH_4_COOH 5 mM and (**b**) NH_4_F 0.2 mM.

CER species with C42 total carbon atoms were selected to represent the seven different subclasses we were able to detect in the studied conditions: Cer[AH], Cer[NH], Cer[AP], Cer[AS], Cer[NP], Cer[NS] and Cer[NDS]. In general, CERs subclasses eluted following the order of the decreasing number of hydroxyl groups in RPLC. The series of 7 CERs with 42 carbon-atoms was chosen to illustrate the one-to-one comparison of the intensity and peak shape of the [M − H]^−^ ion produced by NH_4_COOH and NH_4_F assisted ionization, respectively (Fig. 4).

In general, the [M − H]^−^ ion had an intensity one order of magnitude higher with NH_4_F, while the peak shape was comparably satisfactory. As shown above, it is interesting to observe that when the EIC of the deprotonated ions of Cer[AH] and Cer[AP] was derived from LCMS analyses performed with NH_4_COOH, two peaks were extracted, confirming the need to disambiguate isobaric species in absence of additional information, when using formate salts.

The evidence collected proving NH_4_F as an efficient mobile phase modifier also supports its benefits in DIA to capture all CERs sharing the same diagnostic fragment. The DIA is defined as all-ions acquisition mode with the Q-TOF MS used in our study. The all-ions approach can be useful to detect precursor ions through predominant fragments arising from both the LCB and the FA moieties. Both fragment sources are chain length-specific and double bond-specific. For the all-ions MS/MS acquisitions we set up three parallel fragmentation conditions characterized by different CE, such as 0, 20, and 40 eV, respectively. We used LipidCreator (version 1.1.0) to generate a spectral library in silico and compare acquired MS/MS spectra with the theoretical ones^23^. The assignment of the CERs structure was achieved through the characteristic product ion generated by collision induced dissociation (CID) in negative ESI. It is possible to recognize two groups of fragments attributable to LCB and FA moieties, respectively. In particular, the LCB moiety generates 3 major ions, i.e., LCB(− H6NO), LCB(− CH3O), and LCB(− C2H8NO); the FA moiety gives rise to 3 major fragments, i.e., FA(+ C2H3N), FA(+ HN), and FA(+ C2H3NO). CERs containing α-hydroxy FA, generate a forth fragment, i.e., FA(− CH2O). FA(+ C2H3N) is the most abundant ion fragment. A total of 31 most abundant CERs families in the SC representing each different subclass were identified and characterized using NH_4_F, according to the fragmentation rules extensively reported in the literature for − ESI mode^8,25^. The list of the fragments and annotated CERs subgroups are reported in Supplementary Tables S3 and S4. Empirical masses are represented in the spectral library in silico, while acquired fragmentation spectra display the accurate masses. An example of an identified CER and the related spectral interpretation is reported in Supplementary Fig. S4.

**Figure 5:**
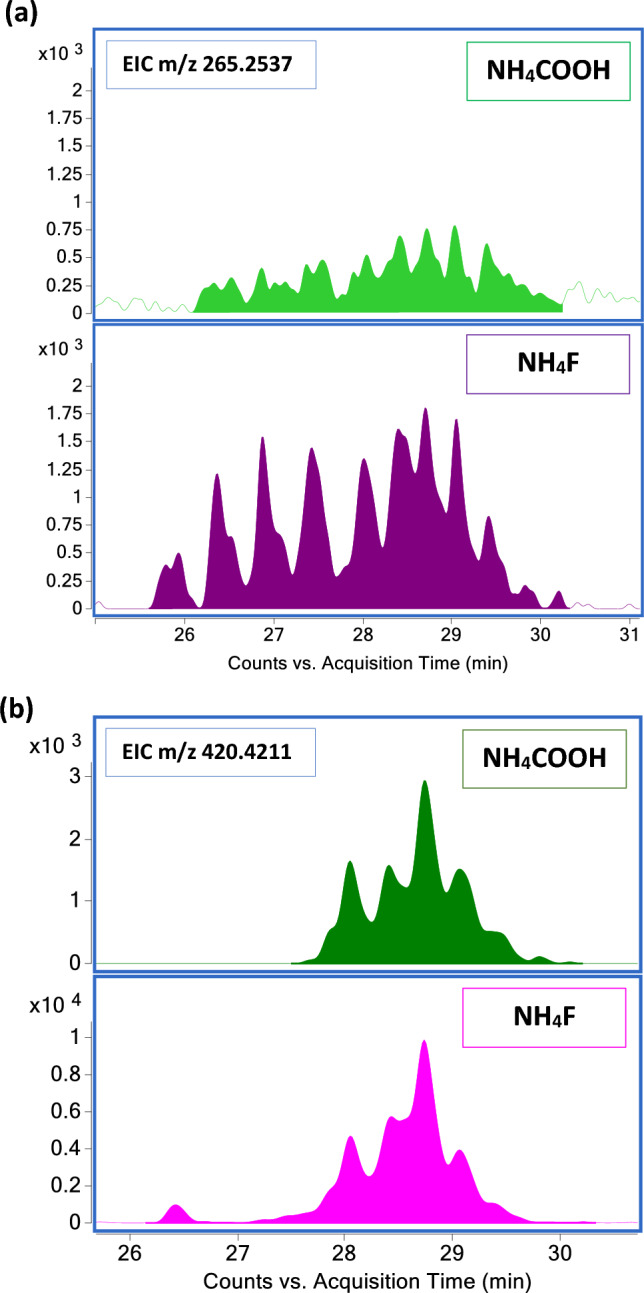
All-ions approach in the comparison of the two mobile phase modifiers. (**a**) EIC of the product ion m/z 265.2537 at a CE of 40 eV from the SC. Comparison of the Cer[NS] LCB C20:1(− C2H8NO) between NH_4_COOH 5 mM and NH_4_F 0.2 mM. (**b**) EIC of the product ion m/z 420.4211 at a CE of 40 eV from the SC sample. Comparison of the FA 26:0(+ C2H3N) between NH_4_COOH 5 mM and NH_4_F 0.2 mM.

To compare the two mobile phase modifiers, the fragment m/z 265.2537, corresponding to the Cer[NS] LCB C20:1(−C2H8NO), was extracted at a CE of 40 eV and integrated from 26 to 30 min (Fig. 5a). The fragment with m/z 420.4211 corresponding to the FA 26:0 (+ C2H3N), was extracted at a CE of 40 eV and integrated from 26 to 30 min (Fig. 5b). In both examples, the enhancing signal effect of NH_4_F is noticeable.

### Ammonium formate versus ammonium fluoride in the LCMS analysis of O-acylceramides

**Figure 6:**
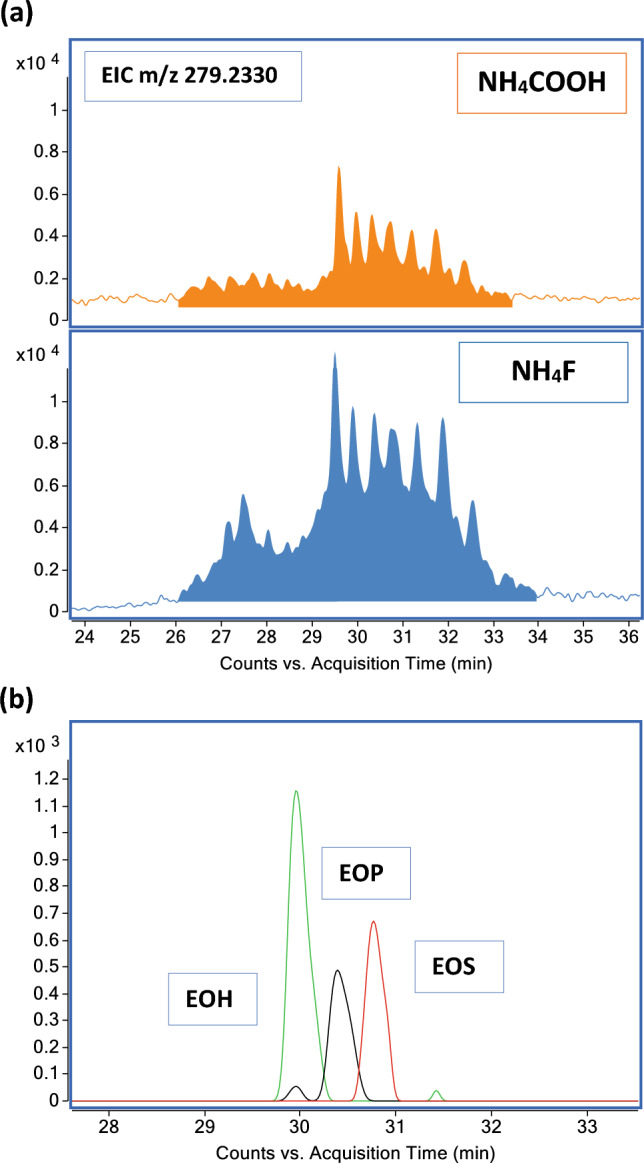
Mobile phase modifiers comparison in the analysis of *O*-acylceramides. (**a**) EIC of the product ion m/z 279.233 (FA 18:2, LA) at a CE of 40 eV from the SC extract. Comparison of the Cer[EO] between NH_4_COOH 5 mM and NH_4_F 200 µM chromatographic conditions. (**b**) Elution order of Cer[EO]. RT were EOS > EOP > EOH.

O-Acylceramides play a significant role in skin homeostasis, ensuring the formation and stabilization of lipid organization in the lipid lamellae^6^. In SC, *O*-ceramides mainly bear ester-linked linoleic acid (LA)^9^. Because of that, linoleoxy-acylceramides were inspected upon extraction of the LA carboxylate anion [M − H]^−^ (m/z 279.233) at a CE of 40 eV from the SC sample^26^. Figure 6a shows a series of peaks eluting in a wide range of RT that represent multiple linoleoyloxy-acylceramides. The quantitative analysis was conducted manually integrating from 26 to 34 min all the species that contain m/z 279.233. The elution order is observable in Fig. 6b, which reports EICs of the EOS, EOP, and EOH containing 66 carbon atoms. NH_4_F enhancing signal effect compared to NH_4_COOH is evident. Fragmentation spectra of d9-CER[EOS] were further tested in NH_4_F conditions (Supplementary Fig. S5).

### Ammonium formate versus ammonium fluoride in the LCMS analysis of free fatty acids and cholesterol sulfate

**Figure 7:**
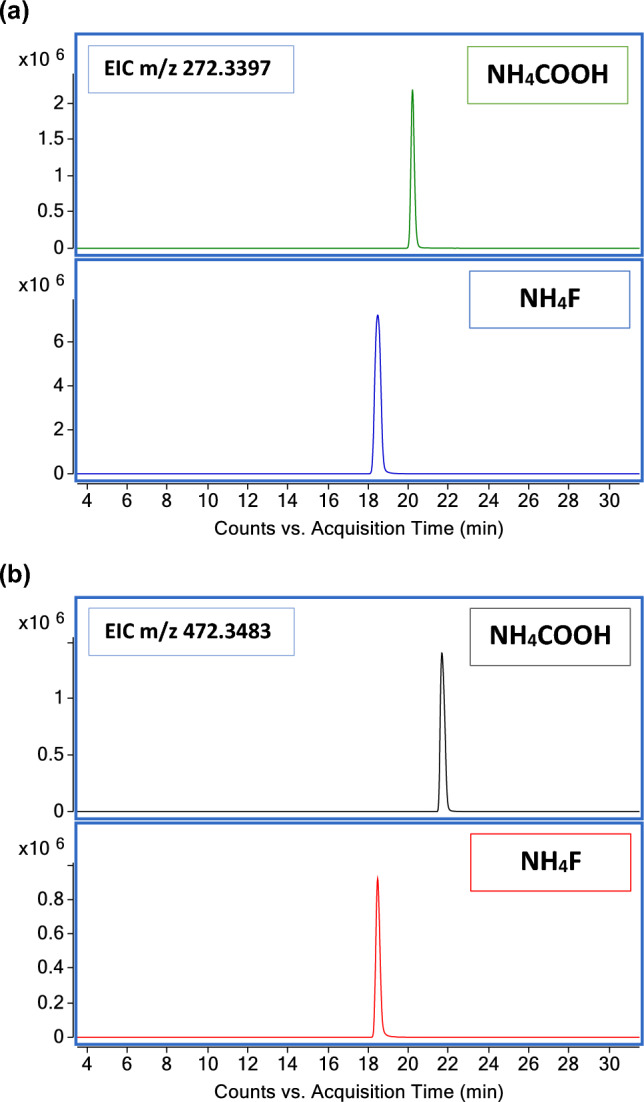
Comparison of mobile phase modifiers in the analysis of the deuterium labelled FFA and CHS. (**a**) Comparison of the peak shape and intensity of the EIC m/z 272.3397 corresponding to the [M − H]^−^ ion of the d17-PA. (**b**) Comparison of the peak shape and intensity of the EIC m/z 472.3483 corresponding to the [M − H]^−^ ion of the d7-CHS.

The ionization efficiency we accomplished with NH_4_F for CERs was tested on FFAs and CHS as major SC components. The two chromatographic conditions were compared using the deuterium-labelled lipids representing each category, i.e., d17-PA and d7-CHS. Figure 7 shows RT and peak shape of d17-PA (a) and d7-CHS (b), in the two studied conditions. Earlier elution was observed with NH_4_F. The effect was more evident in the case of more dissociated acid species such as CHS, and less evident for FFAs. NH_4_F increased the peak height of [M − H]^−^ ion of the d17-PA species. On the other hand, a broadening of the peak shape was observed. Slightly more intense peak of the d7-CHS species was observed with NH_4_COOH, while the peak shape and the peak symmetry were both preserved. The improvements achieved with NH_4_F for CERs were not entirely extendible to CHS.

**Figure 8:**
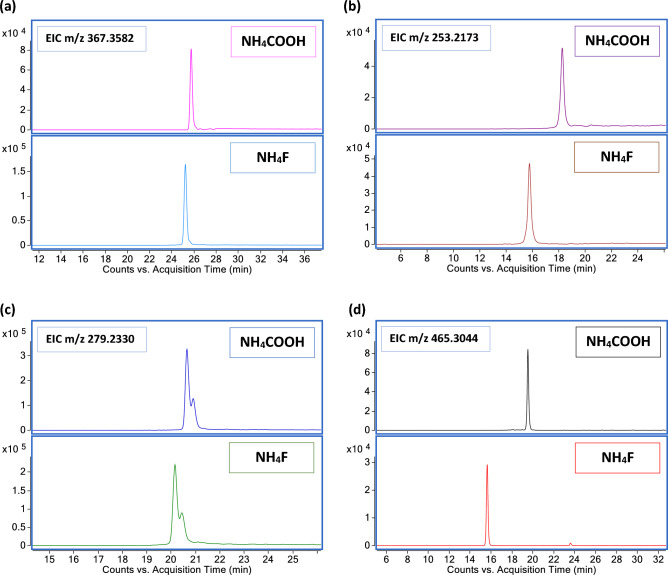
Comparison of mobile phase modifiers in the analysis of FFAs and CHS. (**a**) Comparison of the peak shape and intensity of the EIC m/z 367.3582 corresponding to the [M − H]^−^ ion of FA 24:0. (**b**) Comparison of the peak shape and intensity of the EIC m/z 253.2173 corresponding to the [M − H]^−^ ion of FA 16:1. (**c**) Comparison of the peak shape and intensity of the EIC m/z 279.2330 corresponding to the [M − H]^−^ ion of FA 18:2. (**d**) Comparison of the peak shape and intensity of the EIC m/z 465.3044 corresponding to the [M − H]^−^ ion of CHS.

To investigate the impact of NH_4_F on the detection of epidermal lipids of both categories occurring in the human SC, we conducted a comparison of the two mobile phase modifiers (Fig. 8). We focused on the [M-H]- ions of the FA 24:0 (a), FA 16:1 (b), FA 18:2 (c), and CHS (d). We observed minimal differences on the analytical responses of FFAs and CHS between NH_4_COOH and NH_4_F. However, when using NH_4_F, an earlier RT was observed, as shown above for d17-PA. NH_4_F caused a mild increase of the peak height of [M − H]^−^ ion of the FA 24:0 (Fig. 8a), compared to NH_4_COOH while the effects for FA 16:1 (Fig. 8b) and FA 18:2 (Fig. 8c) were found to be comparable. FA 18:2 was observed in two different isoforms and its abundance was measured by integrating both chromatographic peaks. The first eluted peak was identified as LA (9Z,12Z-octadecadienoic acid), while the second peak was tentatively assigned as sebaleic acid (5Z,8Z octadecadienoic acid) as previously described^27^, due to the occurrence of this isomeric FA 18:2 in human skin lipids. The findings for d7-CHS were applicable to the naturally occurring analyte. Figure 8d shows an earlier elution of CHS when using NH_4_F, while the use of NH_4_COOH favored slightly the peak intensity.

### Application of LCMS conditions using ammonium fluoride to the analysis of pooled extracts of stratum corneum from skin areas rich and poor in sebaceous glands

We analyzed pools of lipid extracts of SC derived from SGR and SGP areas injected repeatedly on several analytical batches. The pmol of each species were derived by multiplying the area ratio of the analyte and the iSTD by the pmol of the iSTD.

To evaluate the repeatability of the amounts and the RTs in the conditions using the NH_4_F modifier, intra-day precision was determined from triplicates of the SGR and SGP pools. Inter-day precision was determined on amounts and RTs determined in SGR and SGP pools analyzed over 17 batches.

**Figure 9:**
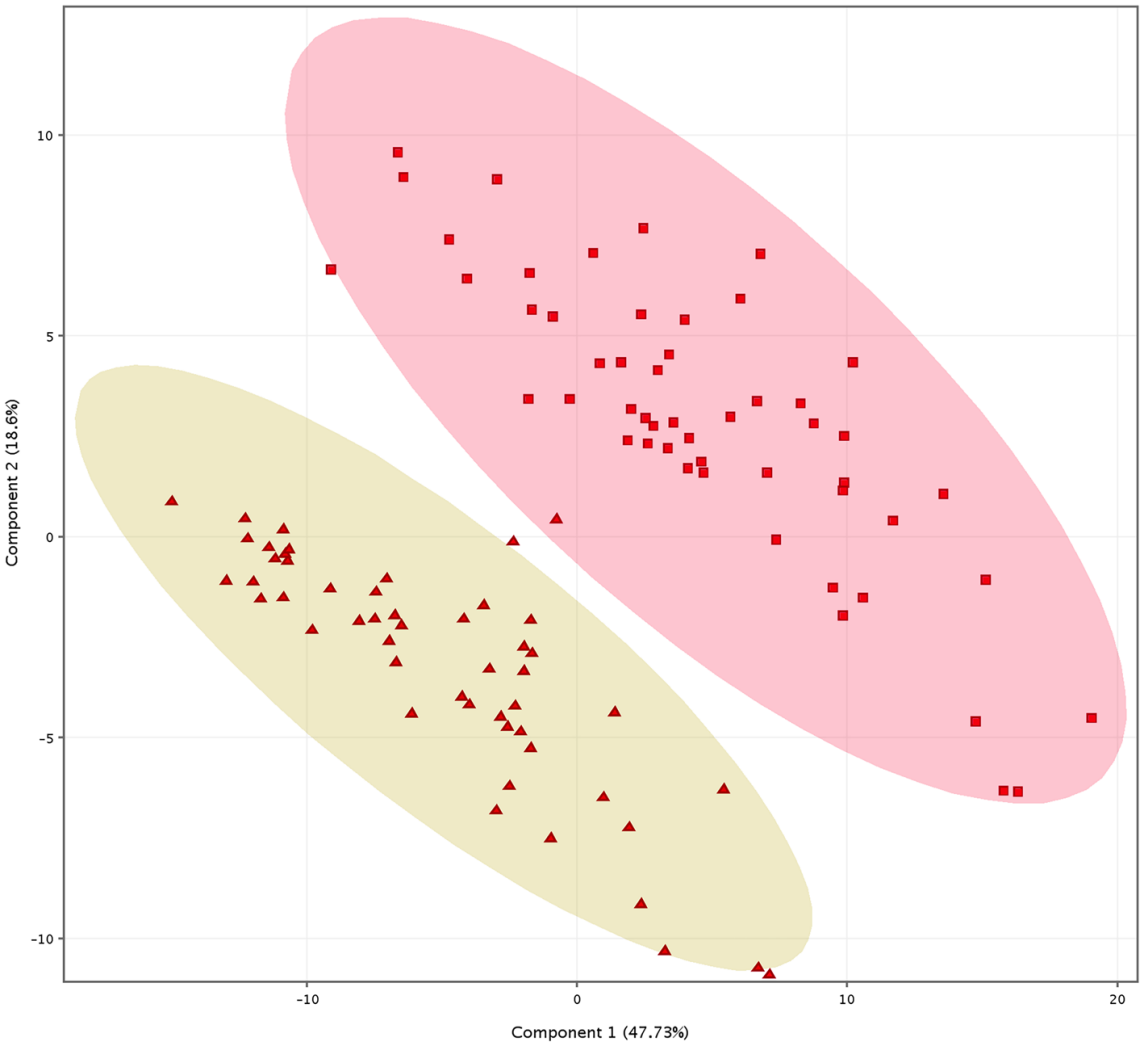
Principal component analysis on extracts of stratum corneum from skin areas rich and poor in sebaceous glands. PCA: Scores plot on the first principal component (PC1) and the second principal component (PC2). The 52 replicates of each pool, i.e. SGR and SGP pools, are projected into the plane spanned by their two PC. The projection shows distinction between samples from SGR and SGP pools, marked with triangles and squares, respectively.

Intra-day and inter-day precision were expressed as coefficient of variation (%CV) and were considered acceptable at %CV ≤ 20 (Supplementary Data 1, 2). In terms of absolute abundance, the species most affected by variations were long chain CERs. In terms of RT, all data were below the %CV threshold. The overall results were more than solid and demonstrated a satisfactory repeatability. To define reproducibility of the injections and individuate differences in the abundance of lipid species detected with the NH_4_F modifier, the pmol obtained for the annotated species were transferred into a template (.csv) and imported into the MPP software. Data in linear scale were log2 transformed before multivariate analysis. The baseline option ‘baseline to mean of samples’ was chosen. After that, samples were divided into groups according to specific parameters to create interpretations. The principal component analysis (PCA) was performed to investigate the consistency of analyses of the same-type samples injected multiple times in different days and to observe the extent of discrimination between the SGR and the SGP pools (Fig. 9). The profiles of abundance of skin lipids in SGR and SGP areas were significantly different, and the first principal component (PC1) represented the 47.73% of the total variability of the data. To better understand the separation effect between the two areas, we inspected the loadings (Supplementary Data 3, 4). Long chain Cer[NDS] having a chain length from 46 to 50 carbon atoms, and very long chain Cer[NS]51:1 and Cer[NS]52:1 showed higher intensity in the SGP pool. Short chain Cer[AS], Cer[AP] and Cer[AH] were more abundant in the SGP pool. Cer[NP] and Cer[NH] were almost equally distributed in SGR and SGP areas. Linoleoxy-acylceramides (CerEO-C18:2), CHS and the analyzed FAs, i.e., FA 24:0, FA 16:1 and FA 18:2, were abundant in in the SGR pool.

## Discussion

The large number of CERs populating the permeability barrier in the SC and their wide-ranging concentration and structural diversity require a suitable method for targeted profiling and characterization of candidate species. Since the other lipids specific to the epidermal compartment, FFAs and CHS, are naturally abundant in the SC matrix, the major focus was the implementation of an analytic process strategic for CERs, which pose challenges due to wide ranges of structures and concentrations. The negative − ESI MS/MS mode provides an effective strategy for the structural comprehensive characterization of sphingolipids^25^. In fact, as compared to the + ESI ion mode, product ions referring to the FA and LCB moieties make the structure of a CER easily deductible^12,14^. Furthermore, − ESI allows for a multiclass analysis that assists the simultaneous determination of the most abundant and representative epidermal lipids^3^. Taking into account these two remarkable advantages, we have optimized an analytical approach that is able to improve the ionization efficiency, the chromatographic separation and the MS detection.

We have compared NH_4_COOH, which is one of the most common modifiers in metabolomic and lipidomic analyses, with NH_4_F, due to its increasing use in LCMS workflows more recently. We have tested the concentration that of NH_4_F that is typically used for hydrophobic compounds^24^. With NH_4_COOH we observed increased complexity and ambiguities linked to the elemental composition of some CERs.

Differently from NH_4_COOH, we observed that NH_4_F promotes the formation of the [M − H]^−^ deprotonated pseudomolecular ion as the dominant one, while the fluoride-anionic adduct [M + F]^−^ is observed with a lower intensity. The conditions favoring the formation of anionic adducts are not fully understood yet, however, in general, the fluoride ions’ strong basicity in the gas phase facilitates the extraction of protons from neutral analytes^22,28^. There is more than one evidence of the efficiency of NH_4_F in proton abstraction when using fluoride-mediated negative ionization in LCMS/MS. For example, NH_4_F providing higher sensitivity and consequently higher level of information, has been used for the in-depth characterization of N-linked oligosaccharides^29^ and for the analysis of the spironolactone^22^. Beyond its capability of increasing sensitivity^30^, NH_4_F proved to enhance peak height, and area, and to improve peak shape of CERs. NH_4_F-assisted ionization also shows improvements in the MS/MS fragmentation where ion fragments have higher SNR, which is advantageous in the structure identification^17^. Generally, the fragmentation at low energy (20 eV) of the [M + X]^−^ anion adduct of CERs produces the preferential loss of the anion. The fundamental structural information referring to the substituent FA and the LCB moieties are almost unobservable when using NH_4_COOH^31^. In our observations, the interpretation of the MS/MS spectra was facilitated when using NH_4_F, especially for low abundant CERs. Moreover, the formation of the adduct with fluoride is easier to identify. The NL of HF (20.0062 Da) is more directly captured compared to the CH2O2 (46.0055 Da) loss.

When increasing the CE at 40 eV, which is a common strategy to promote fragmentation, NH_4_F provides higher diagnostic fragments and increases confidence in the structural assignment compared to NH_4_COOH^18^. Further advantages are provided by the observation that the product ion spectra of the [M + F]^−^ adduct at higher CE is similar to that obtained from the deprotonated precursor ion, although fragments are less intense. This may indicate that upon CID the first step of the decomposition of [M + F]^−^ in the gas phase involves the NL of HF and the subsequent formation of the [M − H]^−^ deprotonated ion^31^. This first fragmentation stage consumes some energy causing lower intensity of the product ions^18^. However, the MS/MS spectrum of the [M − H]^−^ ion is consistently the most informative one. MS/MS spectra of the [M − H]^−^ ion obtained with the NH_4_COOH modifier are comparable to those observed when using NH_4_F, but diagnostic fragment intensity was definitely higher with the latter modifier. Enhanced sensitivity is particularly advantageous when limited sample amounts are available. Tape stripping is a suitable minimally invasive method to collect SC. To overcome the small amounts of SC sampled, multiple patches are usually used to collect sufficient SC amenable for lipid profiling. By increasing the number of extracted patches, the introduction of plastic matter in the LCMS system is rather undesired and sometimes troublesome. Most methods are based on SC collected with 6 to 10 patches to detect CERs^32–34^. In contrast, NH_4_F allows for the satisfactory detection in SC extracts from only two patches. Since NH_4_F enhances signal intensity, it is advantageous for both detection and structural characterization of CERs presenting a wide range of structures and concentrations in the SC. Further advantages arise from the preservation of the detection of FFAs and CHS, which can be analyzed simultaneously with CERs. Using NH_4_F also allowed discrimination between samples of SC derived from areas with different densities of the SGs.

### Supplementary Information

Supplementary Information.

Supplementary Tables.

Supplementary Dataset 1.

Supplementary Dataset 2.

Supplementary Dataset 3–4.

## Abbreviations

A: α-Hydroxy
CE: Collision energy
CERs: Ceramides
CHS: Cholesterol sulfate
DS: Dihydrosphingosine
EIC: Extracted-ion chromatogram
EO: Esterified ω-hydroxy
ESI: Electrospray ionization
FA: Fatty acids
FFAs: Free fatty acids
H: 6-Hydroxysphingosine
HRMS: High resolution mass spectrometry
iSTD: Internal standard
LC: Liquid chromatography
LCB: Long chain base
MS: Mass spectrometry
MS/MS: Tandem mass spectrometry
N: Non-hydroxy
NL: Neutral loss
O: ω-Hydroxy
P: Phytosphingosine
QC: Quality control
Q-TOF: Quadrupole-time of flight
RPLC: Reversed phase LC
RT: Retention time
S: Sphingosine
SC: Stratum corneum
SD: Sphingadienine
SGP: Sebaceous gland poor
SGR: Sebaceous gland rich
SNR: Signal to noise ratio
