# Supplementary material for: Comparison between ammonium formate and ammonium fluoride in the analysis of stratum corneum lipids by reversed phase chromatography coupled with high resolution mass spectrometry
Source: Sci Rep. 2024 Jan 2;14:40. doi: 10.1038/s41598-023-50051-1 (PMC10762128; doi:10.1038/s41598-023-50051-1)
Supplement: Supplementary file 1 — Supplementary Information. [file 41598_2023_50051_MOESM1_ESM.pdf]

Supplementary Information

Supplementary Figure S1

(a)

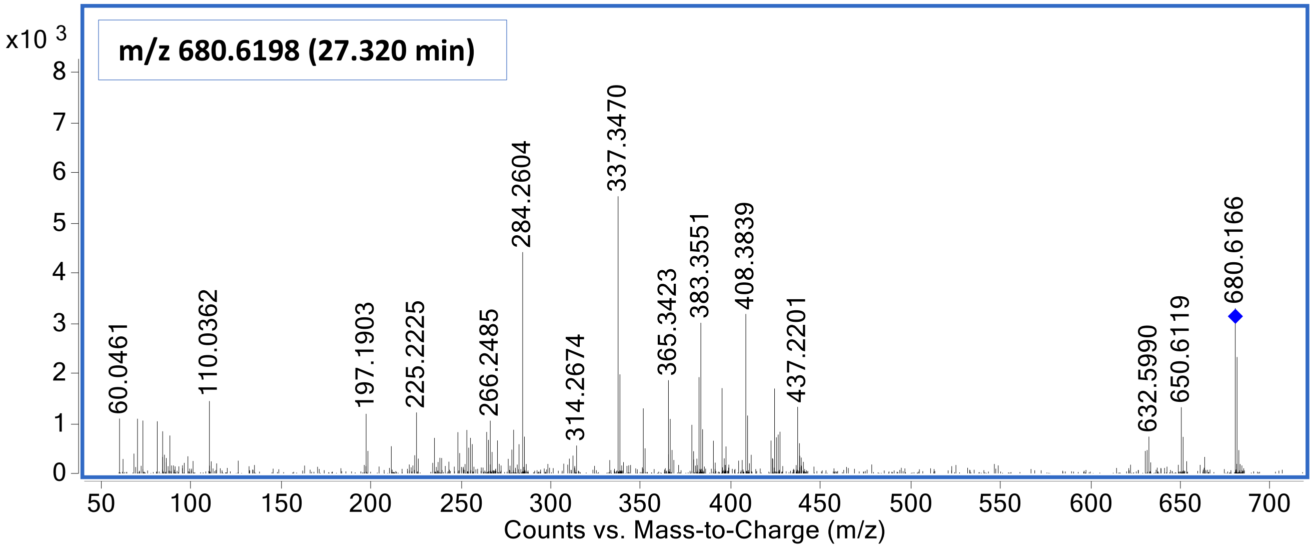

(b)

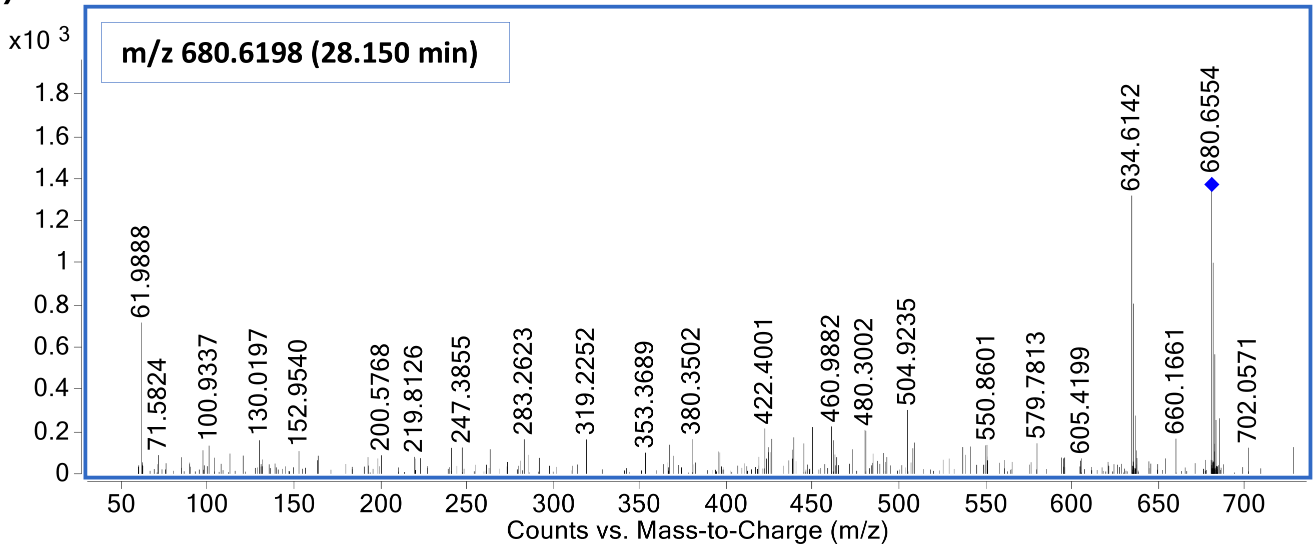

Supplementary Figure S1. MS/MS spectrum at a CE of 40 eV obtained by targeted fragmentation of m/z 680.6198 using NH_4COOH as eluent additive.
(a) Cer[AH]42:1, NH_4COOH assisted ionization $[M-H]^-$ MS/MS spectrum (27.320 min).
(b) Cer[NS]41:1, NH4COOH assisted ionization $[M+HCOO]^-$ MS/MS spectrum (28.150 min).

Supplementary Figure S2

(a)

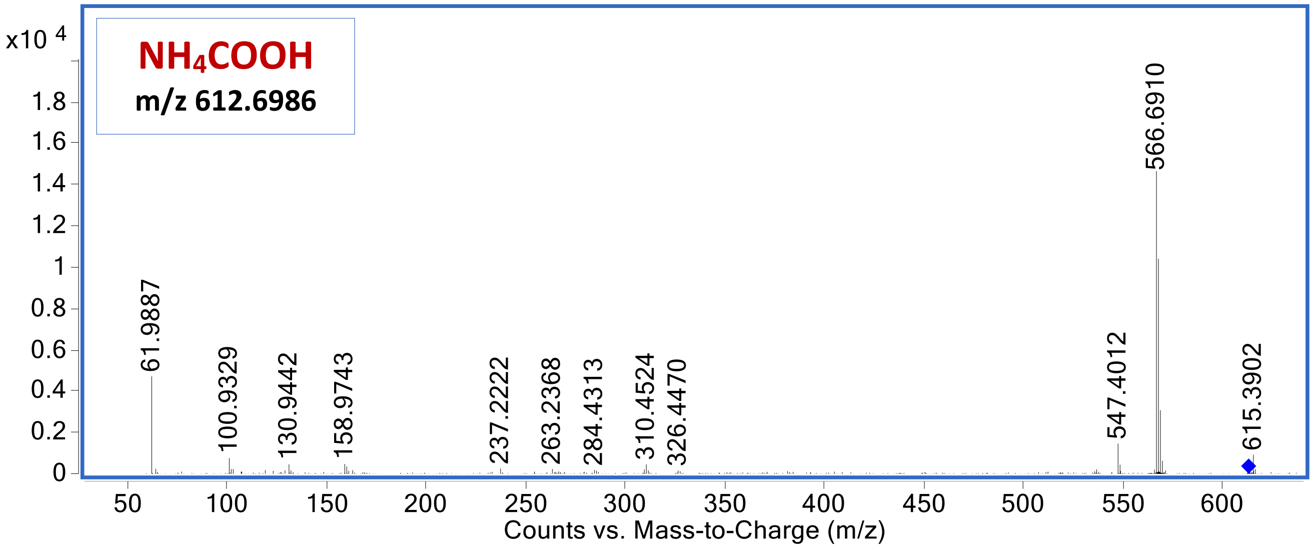

(b)

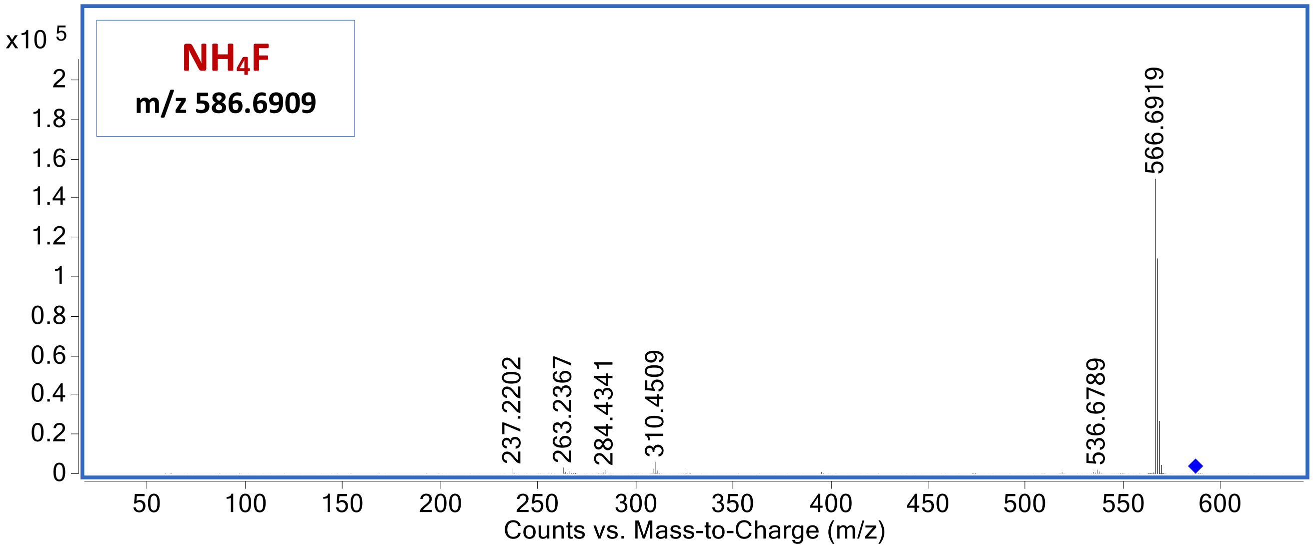

Supplementary Figure S2. MS/MS spectrum at a CE of 20 eV obtained by targeted fragmentation of $[M+X]^-$ anionic adducts of the deuterated ceramide d31-Cer[NS]34:1.
(a) NH_4COOH assisted ionization $[M+HCOO]^-$ MS/MS spectrum.
(b) NH_4F assisted ionization $[M+F]^-$ MS/MS spectrum.

Supplementary Figure S3

(a)

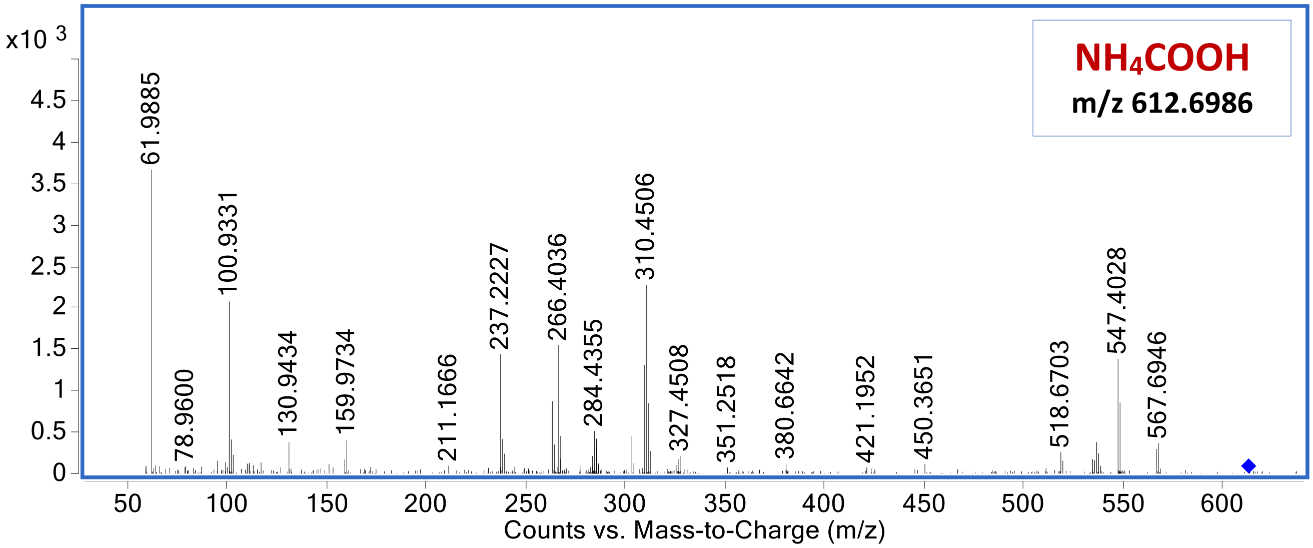

(b)

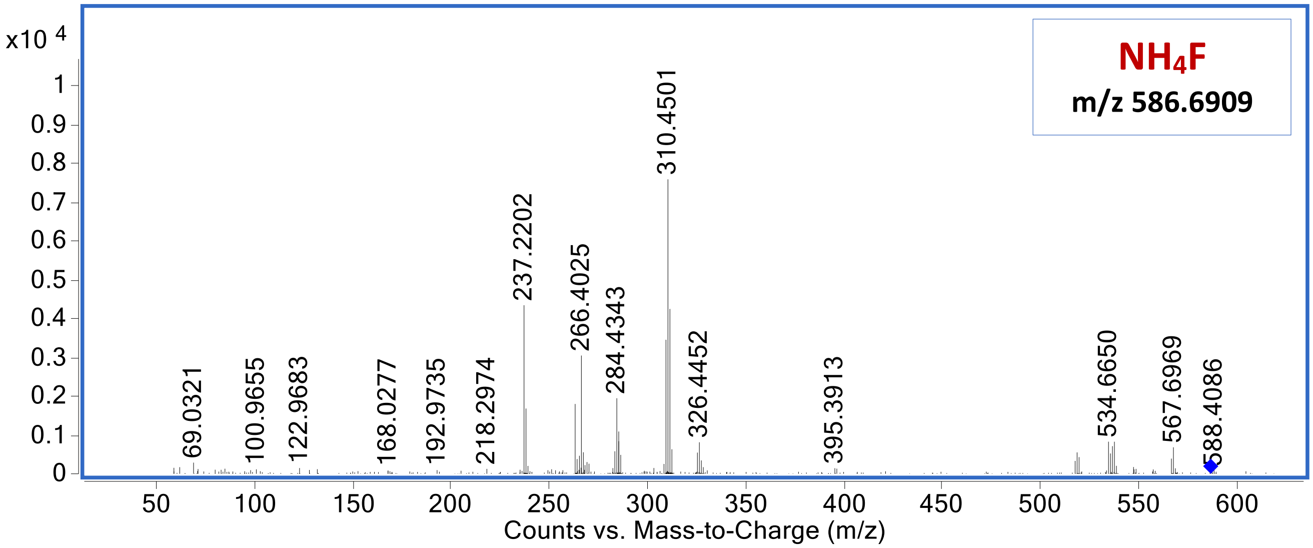

Supplementary Figure S3. MS/MS spectrum at a CE of 40 eV obtained by targeted fragmentation of $[M+X]^-$ anionic adducts of the deuterated ceramide d31-Cer[NS]34:1.
(a) NH_4COOH assisted ionization $[M+HCOO]^-$ MS/MS spectrum.
(b) NH_4F assisted ionization $[M+F]^-$ MS/MS spectrum.

Supplementary Figure S4

(a)

Ceramide	Formula	M	[M-H]⁻	RT
Cer[NS]34:1	C34H67NO3	537.5104	536.5048	26.21

Precursor name	LCB (-H6NO)	LCB (-CH3O)	LCB (-C2H8NO)	FA(+C2H3N)	FA(+HN)	FA (+C2H3NO)
Cer(d18:1/16:0)	263.2380	268.2650	237.2220	280.2650	254.2490	296.2600

(b)

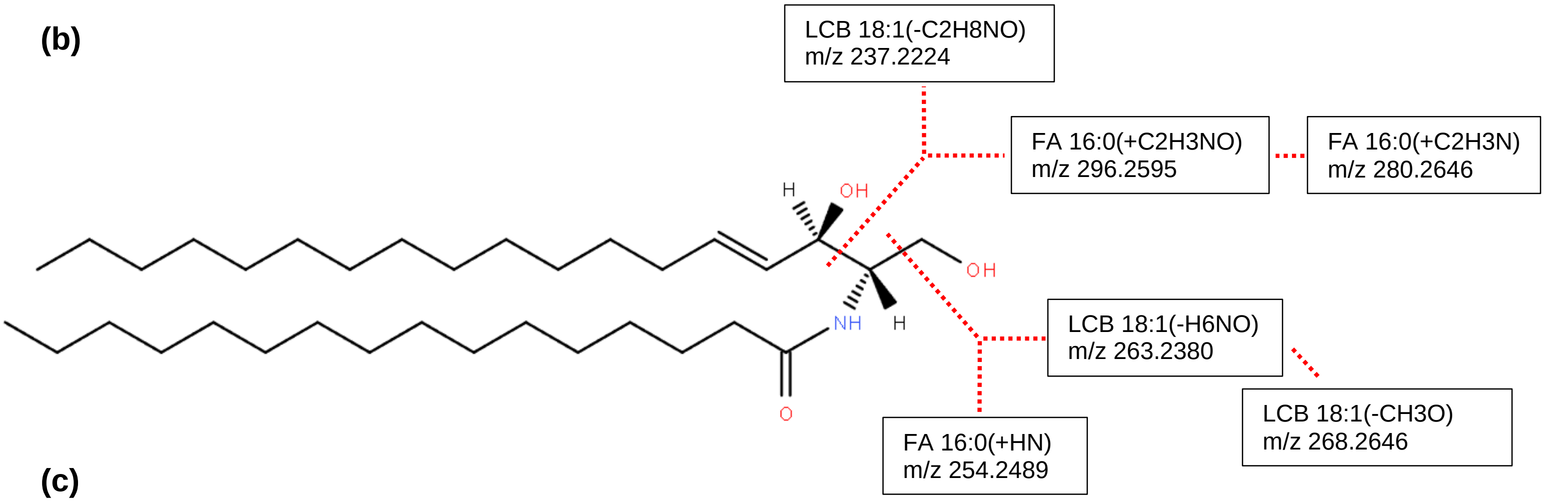

(c)

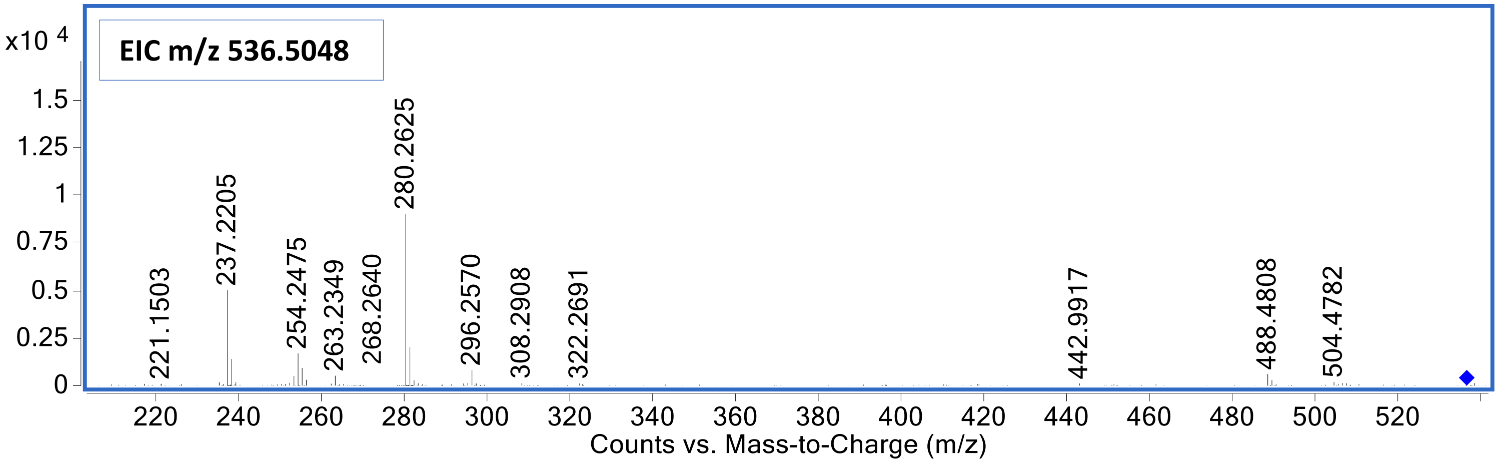

Supplementary Figure S4. Interpretation of the MS/MS spectrum of [M+H]⁻ ion of the identified Cer[NS]34:1, Cer(d18:1/16:0).
(a) List of the fragments generated from LCB and FA of Cer[NS]34:1 by LipidCreator.
(b) Chemical structure and diagnostic fragmentations of LCB and FA of the Cer(d18:1/16:0).
(c) MS/MS spectrum at CE of 40 eV obtained by targeted fragmentation of [M+H]⁻ ion of the Cer(d18:1/16:0) using NH_4F as eluent additive.

Supplementary Figure S5

(a)

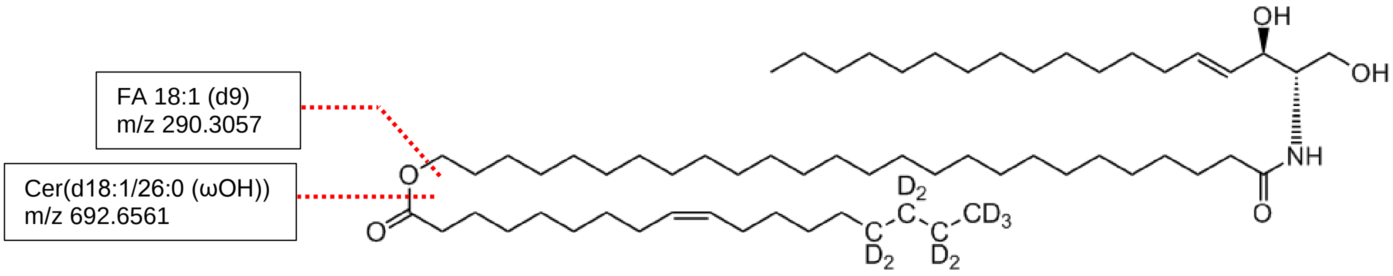

(b)

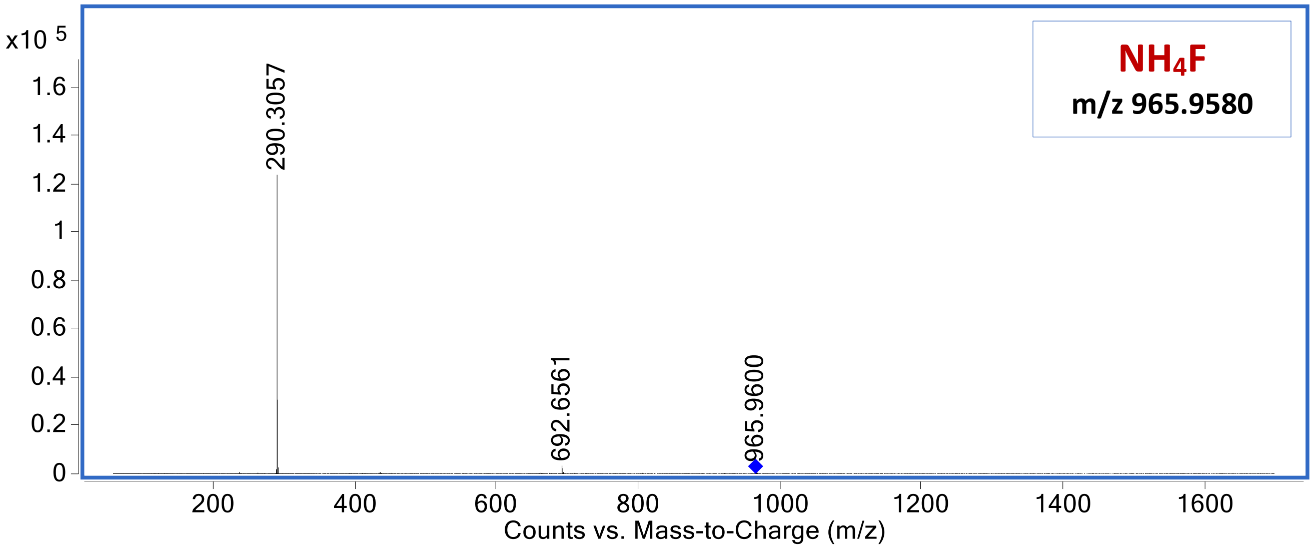

Supplementary Figure S5. MS/MS spectrum at a CE of 40 eV obtained by targeted fragmentation of deprotonated ion of d9-CER[EOS] standard.
(a) Chemical structure and diagnostic fragmentations pattern of the Cer(d18:1/26:0/18:1(d9)).
(b) NH_4F assisted ionization $[M-H]^-$ MS/MS spectrum.

TABLES LEGENDS
(Excel files)

Supplementary Table S1.
The empirical formula and accurate mass of the $[M+HCOO]^-$ and $[M-H]^-$adduct about Cer[AH] and Cer[NS] subclasses.

Supplementary Table S2.
The empirical formula and accurate mass of the $[M+HCOO]^-$ and $[M-H]^-$adduct about Cer[AP] and Cer[NDS] subclasses.

Supplementary Table S3.
List of the fragments and annotated CERs subgroups, Cer[NDS], Cer[NS], Cer[NP], Cer[NH].

Supplementary Table S4.
List of the fragments and annotated CERs subgroups, Cer[AH], Cer[AP], Cer[AS].

Supplementary Data 1.
Intra-day and inter-day precision on pmol amounts determined from triplicates of the SGR and SGP pools analyzed over 17 batches expressed as coefficient of variation (% CV).

Supplementary Data 2.
Intra-day and inter-day precision on RTs determined from triplicates of the SGR and SGP pools analyzed over 17 batches expressed as coefficient of variation (% CV).

Supplementary Data 3-4.
Variable loadings on SC1, SC2, SC3 and SC4 on SGR and SGP areas.
